# Evidence for simultaneous syntactic processing of multiple words during reading

**DOI:** 10.1371/journal.pone.0173720

**Published:** 2017-03-09

**Authors:** Joshua Snell, Martijn Meeter, Jonathan Grainger

**Affiliations:** 1 Aix-Marseille University, Marseille, France; 2 Brain and Language Research Institute, Aix-en-Provence, France; 3 Vrije Universiteit, Amsterdam, The Netherlands; 4 Centre National de Recherche Scientifique, Marseille, France; University of Akron, UNITED STATES

## Abstract

A hotly debated issue in reading research concerns the extent to which readers process parafoveal words, and how parafoveal information might influence foveal word recognition. We investigated syntactic word processing both in sentence reading and in reading isolated foveal words when these were flanked by parafoveal words. In Experiment 1 we found a syntactic parafoveal preview benefit in sentence reading, meaning that fixation durations on target words were decreased when there was a syntactically congruent preview word at the target location (*n*) during the fixation on the pre-target (*n-1*). In Experiment 2 we used a flanker paradigm in which participants had to classify foveal target words as either noun or verb, when those targets were flanked by syntactically congruent or incongruent words (stimulus on-time 170 ms). Lower response times and error rates in the congruent condition suggested that higher-order (syntactic) information can be integrated across foveal and parafoveal words. Although higher-order parafoveal-on-foveal effects have been elusive in sentence reading, results from our flanker paradigm show that the reading system can extract higher-order information from multiple words in a single glance. We propose a model of reading to account for the present findings.

## Introduction

Through decades of reading research, much insight has been gained into how properties of fixated (i.e., foveal) words and upcoming (i.e., parafoveal) words influence eye movement behavior as well as processes of word recognition and sentence comprehension. However, the depth of processing of parafoveal words remains a hotly debated issue. Nevertheless, multiple lines of research have suggested that this is likely to depend on several factors, such as the language at hand (e.g. [1–5]; [6], for a review) and inter-individual differences (e.g. [7]).

There is already considerable evidence that upcoming words are processed sub-lexically in alphabetic languages, and moreover, that orthographic information is integrated across foveal and parafoveal words such that words are recognized faster when they are orthographically related to adjacent words (e.g. [8–14]). However, higher-order processing of upcoming words is more controversial (e.g. [6]). Although one study showed for Chinese that semantic information can be extracted from upcoming words [15], similar investigations have yielded equivocal results for alphabetic languages. Hohenstein, Laubrock and Kliegl [16] reported a semantic parafoveal preview benefit in German sentence reading, using a fast-priming paradigm where parafoveal previews would change into semantically related targets shortly after a fixation on the pre-target. This finding was later replicated by Hohenstein and Kliegl [17] with a standard boundary paradigm, where previews changed into targets during the saccade from the pre-target to the preview/target location. On the other hand, using the same paradigm, Rayner, Schotter and Drieghe [18] could not establish a semantic preview benefit in English sentence reading. Whether upcoming words are semantically processed might thus depend on the language and its users. Interestingly, Schotter [19] did find a semantic preview benefit in English sentence reading when using synonym previews (e.g. *start—begin*) rather than associative previews (e.g. *ready—begin*), suggesting that in some languages finding a higher-order preview benefit may depend on the semantic relationship between preview and target.

### Addressing syntax instead of semantics

Given that research on the semantic access of parafoveal words in sentence reading has yielded equivocal results (but see [17] for a review concluding that the effect is not so controversial), in the current article we turn to a different form of higher-order processing, namely the *syntactic* classification of words. It is clear that throughout decades of reading research, syntactic parafoveal processing has received far less attention than semantic parafoveal processing. One notable exception is a study reporting a morphological preview benefit for syntactically congruent morphemes in Hebrew [1]. In a recent study of our own, we found that the fixation duration on a given word increased if it was followed by a word of the same syntactic category (e.g. *noun noun*, implying an incorrect continuation of the sentence), as compared to a syntactically legal continuation (e.g. *noun verb*) [13]. A possible explanation for this finding is that the upcoming word signaled ‘incorrectness’, leading to a disruption known as saccadic inhibition (see e.g. [20]).

To our knowledge, the only other investigation of syntactic parafoveal processing is a study by Brothers and Traxler [21], the results of which provide further evidence that readers process the syntactic category of upcoming words. They found that English readers were less likely to skip an upcoming word if it violated syntactic rules (e.g. *noun noun*). However, they did not find the syntactic equivalent of the semantic preview benefit as reported by Hohenstein and Kliegl [17] and Schotter [19]; that is, fixation durations on target words were not increased after syntactically invalid previews, as compared to syntactically valid previews (preview effects were found when the valid preview was a repetition of the target word, but these effects were likely to be orthographic in nature). Furthermore, unlike Snell et al., Brothers and Traxler did not find increased fixation durations on the pre-target word (*n-1*) when the preview (*n*) was syntactically invalid.

Under which conditions syntactic parafoveal-on-foveal effects occur is thus not yet clear, but it seems likely, at least, that these effects are different from the sub-lexical parafoveal-on-foveal effects discussed above, in the sense that they are not facilitatory in nature (e.g., such that processing of a noun type would be sped up by an adjacent noun type). While this may seem logical, it must be noted that previous studies have dismissed the possibility of higher-order parallel processing precisely on the basis of an absence of semantic parafoveal-on-foveal integration (see e.g. [8]). Here we argue, however, that parallel processing does not equal parafoveal-on-foveal integration. Instead, we propose that the brain can keep track of which word has what role in the sentence being read, meaning that higher-order information is kept separate, rather than being integrated, across words. For example, based on sentence constraints we often know that the upcoming word should be a noun, or that a verb will appear two positions to the right. Furthermore, readers can very accurately make regressions to those points in sentences that are critical for resolving syntactic ambiguity (e.g. [22]), suggesting that some representation of the syntactic structure of a sentence is retained in memory (see [23], for a discussion of the role of syntax in parallel word processing).

It is not inconceivable, then, that the reading process is perturbed when the words that are being recognized are syntactically incoherent, explaining why effects of higher-order parafoveal-foveal integration (e.g. lexical, semantic; [13] and [8], respectively) in sentence reading have been elusive. At the same time, in light of this scenario we may predict that higher-order parafoveal-on-foveal integration might take place in a setting where readers do not set out to read sentences, that is, a setting where readers do not create sentence-level representations. One example of such a setting would be a flanker paradigm similar to that used in the studies of Dare and Shillcock [9], Grainger et al. [10] and Snell et al. [13], but now using syntactically related flankers rather than orthographically related flankers.

Here we report two experiments that test the hypothesis that words are syntactically categorized in the parafovea, and that are aimed at further exploring the nature of higher-order parallel processing. In Experiment 1, we used the gaze-contingent boundary paradigm [24] to see if the recognition of a target word (*n*) would be facilitated by a syntactically congruent preview at the target location when readers were fixating the pre-target (*n-1*). In Experiment 2, we used a flanker paradigm in which participants had to indicate in each trial whether a foveally presented target word was noun or verb, while it was flanked by parafoveal words that were syntactically congruent / incongruent with the target (e.g. *cloud horse cloud* vs. *kneel horse kneel*), or words that formed a correct / incorrect sentence with the target (e.g. *young horse jumps* vs. *jumps horse young*).

## Experiment 1: Syntactic preview benefit in sentence reading

### Methods

#### Ethics statement

Given that Experiment 1 and Experiment 2 consisted of non-invasive, low-demanding behavioral experiments, ethical approval was deemed unnecessary. Nevertheless, all participants gave written informed consent to their participation in this study. Participants were further given the option to opt out of the study, but none of the participants made use of this option. For administrative (payment) purposes, participants gave their name, address and student number. The experiments were carried out by the authors of this work.

#### Participants

30 students (19 female, age 18‒26) from the VU University (Amsterdam) participated in this study for €4,- or its equivalent in course credit. All participants were native Dutch speakers and had normal or corrected-to-normal vision. All participants reported to be non-dyslexic. Further, all participants were naïve to the purpose of the experiment.

#### Materials

From the *Dutch Lexicon Project* lexicon [25] we retrieved 150 5-letter target words and 150 5-letter preview words that were noun or verb (75 occurrences of each). Every target was paired with two previews, one of which was syntactically congruent with the target and one of which was incongruent. All previews were thus used twice; once in a congruent condition and once in an incongruent condition. The amount of orthographic overlap with the target was equal for the two preview types, at an average of one letter. We further assigned a 5-letter pre-target to every target, with the rule that the congruent preview would also be a syntactically correct follow-up of this pre-target, whereas the incongruent preview would be an incorrect follow-up. Specifically, when the pre-target was a noun (‘*horse’*), the incongruent preview was also a noun (*‘table’*) and the congruent preview a verb (‘*bites*’). When the pre-target was an adjective or verb (‘*great’* or ‘*bites’*), the incongruent preview was a verb (‘*walks’*) and the congruent preview a noun (‘*grass*’). As such we had the possibility to not only find a preview benefit in the fixation on the target, but also saccadic inhibition during the fixation on the pre-target, replicating the finding of Snell et al. [13] discussed above.

A sentence varying between 29 and 57 characters (including spaces) was constructed for every target. All pre-targets and previews had a lexical decision time (LDT) value between 500 ms and 650 ms, and within each sentence the correct and incorrect preview had an equal LDT.

**Fig 1:**
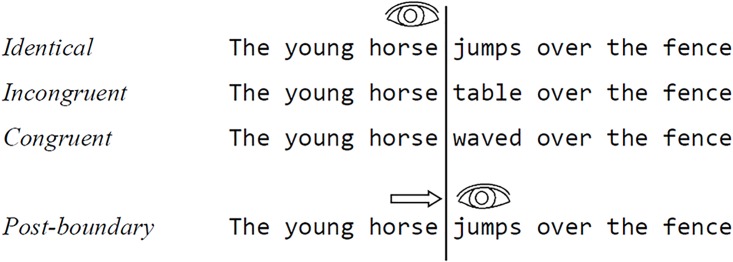
Experiment 1 condition examples. The upper three sentences show what a stimulus could look like in three conditions *before* the eyes cross the boundary (vertical line). We used an identical condition (with target ‘*jumps*’ already visible prior to its fixation), a condition with an incongruent preview (‘*table*’) and a condition with a congruent preview (‘*waved*’). As soon as the eyes move beyond the boundary, the preview changed into the target.

We used the gaze-contingent boundary technique [24] to manipulate the identity of the preview while participants focused on the pre-target. Using an eye-tracker to carefully track the eye position, we changed the preview into the target as soon as an eye movement was made from the pre-target to the preview/target location, (see Fig 1). Besides the congruent and incongruent condition, there was also an identical condition where the target was presented throughout the trial.

To check that participants read for meaning, we created a ‘quiz-question’ for one out of every five items (totaling 10 per condition). Each question was displayed immediately after participants had finished the sentence that it belonged to, and was to be answered with a left / right button response; (two possible answers were displayed in the left- and right-bottom corner of the screen, with the side of the correct answer randomized). All text was displayed in black on a light-grey background, and all stimuli were presented in randomized order.

#### Design

We used a Latin square design to present all stimuli in all three conditions (*congruent preview*, *incongruent preview*, *baseline*), but only once per participant. Thus, for each participant, there were 50 items per condition, amounting to 150 experimental trials which were presented in random order.

#### Apparatus and software

The stimuli and experimental design were implemented with OpenSesame [26], with the PyGaze back-end [27] to process eye movement data online. The participant’s right eye position was recorded with an EyeLink 1000 (SR Research, Mississauga, ON, Canada), a video-based eye tracker sampling at 1000 Hz with a spatial resolution of 0.01°. Stimuli were presented on a 1024x768 px, 150 Hz computer monitor. Participants were seated at a distance of 90 cm from the display, so that each character space subtended 0.35 degrees of visual angle. A chin-rest was used to facilitate a stable head position.

#### Procedure

Before commencing the experiment, the right eye was calibrated using a 9-point calibration grid with fixation points appearing in random order. In case of a sufficient match between the calibration grid and fixation grid, a validation was carried out to double-check the accuracy of the initial fixations. Prior to the actual experiment, a set of five practice trials (including a catch question) was used to allow the participant to become acquainted with the procedure.

At the start of each trial, a drift correction dot was shown in the center of the screen, on which participants had to fixate before pressing the spacebar. This allowed for an automatic drift correction before the start of every trial—and in the case of failing to align the eye with the fixation point, the initiation of a full recalibration.

In case of a successful alignment, a fixation mark in the shape of a forward slash (/) was presented on half a sentence’s length to the left of the screen center. When the eyes had stabilized on this fixation mark (within a 40 pixel range) for 700 ms, the experimental sentence appeared with its center aligned to the center of the screen, meaning that the beginning of the sentence was aligned to the fixation position.

The position of the eyes was tracked online as participants read the sentence. When the eyes moved beyond the pre-target, the preview changed into the target. This display was kept onscreen until the eyes had reached the end of the sentence (with a maximum distance of 30 pixels to the left of the last character of the sentence). A green dot was displayed a little to the right of the sentence’s end when this end was reached. Shortly thereafter, the drift correction screen was displayed again to begin the next trial. However, for sentences with a comprehension question (30 out of 150 trials), the quiz display was presented first, with the two possible answers displayed below in the left / right corner, contingent with the left / right button response. A random side was chosen for the correct answer every trial. The response was met with a green ‘*goed*!’ (*good* in Dutch) or red ‘*fout*!’ (*wrong* in Dutch) message, depending on whether the answer was correct or incorrect respectively. Shortly hereafter, the screen was cleared to start the next trial.

Participants were encouraged to blink before fixating on the slash mark, and to not blink during the presentation of a sentence, because the temporary loss of corneal reflection would be misinterpreted by the eye-tracker (i.e., it would temporarily pass on wrong fixation coordinates to the computer, causing for instance a premature boundary change).

The experiment lasted approximately 25 minutes. After the experiment, a short debriefing was carried out to check if participants noticed any display changes.

### Results

From the total of 4500 trials, 493 trials (10.96%) were discarded due to eye-blinking or the occurrence of a display-change during a fixation (e.g., due to landing too close to the boundary). As all participants answered more than 90% of the catch trials correctly, no participant was excluded. From the 30 participants, 26 reported to have seen a display change. One of these participants reported to have seen a display change at least ten times; all the other participants reported to have seen the display change two or three times.

From the eye tracker data we computed the first fixation duration (FFD), gaze duration (GD) and total viewing time (TVT) on the pre-target and target. Here, FFD refers to the mean first fixation duration on a word, regardless of whether there were subsequent fixations. GD refers to the mean sum of all fixation durations during first pass, i.e., excluding fixation times following a regression back to the word. TVT refers to the mean sum of all fixation durations on a word, both during first pass and following a regression. We further calculated the skipping probability, the probability for a refixation during first pass, and lastly, the probability for a refixation by means of an inter-word regression.

For the duration measures we used linear mixed-effect models (LMMs) with items and participants as crossed random effects [28]. We followed the procedure suggested by Barr, Levy, Scheepers and Tily [29] to determine the maximal random effects structure permitted by the data. This led us to include by-item and by-participant random intercepts for probability analyses, and by-participant random slopes alongside by-item and by-participant intercepts for the analyses of duration measures. The models were fitted with the lmer function from the lme4 package [30] in the R statistical computing environment. We report regression coefficients (*b*), standard errors (SE) and *t-*values. Fixed effects were deemed reliable if | *t* | > 1.96 [28]. Logistic LMMs (fitted with the glmer function) were used to analyze the skipping, refixation and regression probabilities. Here, fixed effects were deemed reliable if | *z* | > 1.96. In all analyses, values beyond 2.5 SD from the mean, (on average 1.7% of the trials), were marked as outliers and excluded.

#### Pre-target fixations

In our previous study we found that the fixation duration on word *n* was increased with a syntactically similar word at position *n+1*, as compared to a condition where word *n+1* was a normal follow-up of *n* [13]. Based on this finding, we expected for the current experiment that the syntactically incongruent preview would lead to increased pre-target fixation durations as compared to the congruent preview, due to saccadic inhibition.

As it turned out, we did not find a significant difference between the congruent and incongruent preview conditions (Tables 1 and 2). A likely cause for this discrepancy is that the fixation durations were in general much lower in the current study as compared to our previous study (mean FFDs of 209 ms and 253 ms, respectively; see the Discussion for a possible explanation of this difference).

**Table 1:** Pre-target means.

	*FFD*	*GD*	*TVT*	*Skip*	*Refix*	*Regress*
*Congruent*	203.8 (72.1)	256.4 (124.5)	291.9 (163.8)	0.12 (0.14)	0.33 (0.26)	0.03 (0.07)
*Incongruent*	207.2 (76.6)	259.7 (124.1)	299.6 (171.6)	0.12 (0.13)	0.31 (0.24)	0.03 (0.07)
*Identical*	203.3 (72.1)	254.6 (121.9)	268.3 (139.4)	0.12 (0.11)	0.31 (0.24)	0.02 (0.07)

Note: Mean fixation durations (ms) and probabilities per condition. Values in parentheses indicate standard deviations. Abbreviations: FFD, first fixation duration; GD, gaze duration; TVT, total viewing time.

**Table 2:** Pre-target duration measures analyses.

	*FFD*			*GD*			*TVT*		
	*b*	*SE*	*t*	*b*	*SE*	*t*	*b*	*SE*	*t*
*(Intercept)*	200.53	5.62	**35.68**	256.66	10.59	**24.23**	293.74	13.46	**21.83**
*Incongruent* ^a^	3.66	2.38	1.54	3.31	4.57	0.72	5.89	5.72	1.03
*Identical* ^a^	0.25	2.39	0.10	-0.38	4.57	-0.08	20.06	5.71	**-3.52**

Note:

^a^ ref.: congruent preview. Significant values are indicated in bold. Abbreviations: SE, standard error; FFD, first fixation duration; GD, gaze duration; TVT, total viewing time.

For the pre-target there was no significant difference in the skipping and refixation rates among conditions (Tables 1 and 3). The regression rate was increased for the congruent and incongruent condition as compared to the identical condition (with *b* = 1.02, SE = 0.15, *z* = 6.97 for the congruent condition and *b =* 1.17, SE = 0.14, *z* = 8.09 for the incongruent condition).

**Table 3:** Pre-target probability measures analyses.

	*Skip*			*Refix*			*Regress*		
	*b*	*SE*	*z*	*b*	*SE*	*z*	*b*	*SE*	*z*
*(Intercept)*	-2.45	0.21	**-11.64**	-1.46	0.20	**-7.19**	-2.07	0.16	**-12.58**
*Incongruent* ^a^	-0.09	0.12	-0.75	-0.06	0.09	-0.66	0.17	0.11	1.57
*Identical* ^a^	-0.08	0.12	-0.62	0.00	0.09	0.01	-0.76	0.13	**-6.07**

Note:

^a^ ref.: congruent preview. Significant values are indicated in bold. Abbreviations: SE, standard error.

#### Target fixations

We expected that a syntactically congruent preview at the target location during the fixation on the pre-target would yield a preview benefit during subsequent target processing. This hypothesis was confirmed, as all fixation duration measures except TVT were significantly increased after an incongruent preview as compared to a congruent preview (with marginal significance for TVT; Tables 4 and 5). All fixation durations were also significantly lower for the identical condition as compared to the two preview conditions. This was to be expected, as the target was already visible during the fixation on the pre-target in the identical condition. Nonetheless, our results show that the cost of having a different word at the target location prior to its fixation, is smaller when that word is syntactically compatible with the sentence.

**Table 4:** Target means.

	*FFD*	*GD*	*TVT*	*Skip*	*Refix*	*Regress*
*Congruent*	239.8 (93.9)	269.3 (122.5)	312.2 (149.4)	0.14 (0.16)	0.14 (0.11)	0.04 (0.05)
*Incongruent*	249.6 (96.0)	284.9 (128.8)	320.8 (156.5)	0.12 (0.16)	0.15 (0.12)	0.04 (0.05)
*Identical*	220.9 (79.6)	241.3 (99.1)	260.0 (119.2)	0.15 (0.16)	0.10 (0.10)	0.03 (0.03)

Note: Mean fixation durations (ms) and probabilities per condition. Values in parentheses indicate standard deviations. Abbreviations: FFD, first fixation duration; GD, gaze duration; TVT, total viewing time.

**Table 5:** Target duration measures analyses.

	*FFD*			*GD*			*TVT*		
	*b*	*SE*	*t*	*b*	*SE*	*t*	*b*	*SE*	*t*
*(Intercept)*	208.46	11.62	**17.94**	230.24	13.31	**17.30**	268.02	16.71	**16.04**
*Incongruent* ^a^	16.75	4.99	**3.36**	19.73	5.42	**3.64**	10.90	6.14	1.78
*Identical* ^a^	-21.42	4.77	**-4.49**	-28.30	5.59	**-5.06**	-51.38	7.91	**-6.50**

Note:

^a^ ref.: congruent preview. Significant values are indicated in bold. Abbreviations: SE, standard error; FFD, first fixation duration; GD, gaze duration; TVT, total viewing time.

Furthermore, replicating the results of Brothers and Traxler [21], we found that the target skipping rate was significantly higher after a congruent preview than after an incongruent preview (Tables 4 and 6). This fits quite well with our hypothesis, as it suggests that the incongruent preview signaled that something was wrong at the target location, prompting more fixations to this location (see also [31]).

**Table 6:** Target probability measures analyses.

	*Skip*			*Refix*			*Regress*		
	*b*	*SE*	*z*	*b*	*SE*	*z*	*b*	*SE*	*z*
*(Intercept)*	-2.35	0.25	**-9.38**	-2.18	0.18	**-12.17**	-1.55	0.12	**-13.43**
*Incongruent* ^a^	-0.40	0.14	**-2.89**	0.17	0.12	1.45	-0.16	0.10	-1.58
*Identical* ^a^	0.18	0.13	1.15	-0.40	0.13	**-3.16**	-0.98	0.12	**-8.15**

Note:

^a^ ref.: congruent preview. Significant values are indicated in bold. Abbreviations: SE, standard error.

### Discussion

The results from Experiment 1 suggest that readers can acquire syntactical information from upcoming words during sentence reading, as all first pass fixation duration measures on the target were decreased when it was preceded by a syntactically congruent preview during the fixation on the pre-target. We also expected increased fixation durations on the pre-target in the incorrect / incongruent condition, caused by a disruption by the incorrect parafoveal preview. Although we found that pre-target fixation durations were indeed numerically increased in this condition, this effect did not reach significance (*b =* 3.66, SE = 2.38, *t =* 1.54), contrary to our previous study [13]. This could have been because fixations were generally shorter in the current study. This difference in FFD might have been caused by the fact that we used 5-letter words in Experiment 1, whereas we used 4-letter words in Snell et al. [13]. The longer word length led participants to sometimes make two short fixations instead of a single longer fixation, as evidenced by the fact that approximately one third of the words were refixated in Experiment 1 (Table 1), as compared to approximately 7% in our previous study. Yet, we also found that targets were skipped more often after a congruent (correct) preview than after an incongruent (incorrect) preview. In line with our hypothesis, this finding suggests that the incorrect preview signaled to readers that something was wrong, prompting more eye-movements to its location. It is further evident that the parafoveal preview could be processed syntactically during the short time window of the pre-target fixation, as we found a clear preview effect at the target location.

As was mentioned in the introduction, Brothers and Traxler did not find a syntactic preview benefit in their study [21]. Drawing an analogy to the equivocal results generated by investigations of the semantic parafoveal preview benefit (e.g. [17–19]), it may be the case that higher-order preview effects—at least in fixation times–are more stable in Dutch and German than in English. At the same time it should not be forgotten that Brothers and Traxler did find increased target skipping rates after syntactically valid previews as compared to invalid previews [21]. Hence, while parafoveal processing effects might manifest themselves differently across languages, the increasing body of results is consistent with the view that higher-order processing can occur for parafoveal words during reading.

As Experiment 1 results provided evidence for higher-order processing of parafoveal words, we set out to investigate the time-course of these processes in Experiment 2 –in particular with respect to whether higher-order processing of parafoveal words may occur *during* or *after* foveal word processing. Indeed, a highly debated issue in reading research concerns the question whether lexical processing can occur across multiple words simultaneously (e.g. [32–34]). While higher-order parafoveal preview effects show that upcoming words can be processed lexically, it has been argued by Schotter, Lee, Reiderman and Rayner that such a finding can still be reconciled with a serial processing account if one assumes that attention moves, ahead of the eyes, to word *n+1* when word *n* is recognized to a certain extent [35]. From that moment on, processing of word *n+1* may allow for lexical access—although it must be acknowledged that the time window within which that should happen is considerably short under the assumption of serial processing, considering that the largest portion of the fixation duration would already be spent on the processing of word *n*.

A more effective measure to assess parallel processing may be that of *parafoveal-on-foveal* information integration. It has already been shown in multiple studies that foveal words are recognized faster when they are surrounded by orthographically related information, both in single word reading and in sentence reading [8–13], indicating that sub-lexical processing occurs across multiple words in parallel. However, as was stated in the introduction, higher-order parafoveal-on-foveal effects are more controversial. In their study, Angele et al. did not find that foveal words were recognized faster when they were semantically related to upcoming words [8]. Similarly, in the current study we did not find that foveal words were recognized faster when they were syntactically related to upcoming words. Indeed, from a theoretical standpoint it can be argued that the reading system would not benefit from integrating syntactical information across multiple words: rather, readers would have to keep track of which word has what role in a sentence in order to understand it properly, implying a fairly strict separation of the multiple word identities it contains.

On the other hand, it can be argued that the syntactic categorization of words may be influenced by sentence-level constraints (see e.g. [36]). Based on the first part of a sentence, for example, readers may have clear expectations about upcoming words, both semantically and syntactically. It is possible that the syntactic recognition of word *n+1* constrains processing of word *n* in a similar way. In this sense, higher-order information integration would not entail the gathering of all available syntactic or semantic information into a single mixture, but rather the construction of a sentence-level representation that interacts with its constituent word identities through feedback connections—a process that can be fundamentally harmonized with a parallel processing account.

In Experiment 2 we employed a flanker paradigm to find out, firstly, whether higher-order (syntactic) information could be integrated across multiple words in parallel, and secondly, whether the nature of this integration process would be one that relies on sentence-level constraints, or one that relies on the integration of syntactic information in a more general sense. To this end, we presented target words in the fovea that were either noun or verb, flanked by words on the left and right which corresponded to either one of four conditions: two conditions where target and flankers would form a grammatically correct or incorrect sentence respectively (to test the hypothesis of sentence-level constraints), and two conditions where target and flankers were syntactically congruent or incongruent (to test the hypothesis of general information integration). As participants had to indicate on each trial whether they read a noun or verb, the expectation was that response times would increase for the incorrect / incongruent flankers as compared to the correct / congruent flankers.

## Experiment 2: Syntactic paraoveal-on-foveal influences

### Methods

#### Participants

22 students (16 female, age 18‒23) from the VU University (Amsterdam) gave written informed consent to participate in this study, carried out by the authors at the VU University. Participants earned €4,- or its equivalent in course credit for their participation. None of these participants had participated in Experiment 1. Participation criteria were similar to those used in Experiment 1. As in Experiment 1, participants had the option to opt out of the study, but none did so.

#### Materials

From the *Dutch Lexicon Project* lexicon [25] we retrieved 50 noun targets and 50 verb targets with a length of 4 or 5 letters (39 and 61 occurrences respectively), from an LDT range of 550–750 ms. Each target was coupled to a syntactically congruent flanker and an incongruent flanker with an equal LDT value. Both flankers were of the same length as the target and had no orthographic overlap with the target. For every target we further chose two words with an equal length ranging between 3 and 5 letters, that would form a correct sentence with the target when the one flanker was on the left and the other on the right (e.g. *young horse jumps*), and an incorrect sentence when the flankers would be switched around (*jumps horse young*).

#### Design

There were four experimental conditions, two of which would test for an effect of syntactical congruency (*congruent* vs. *incongruent* flankers), and two of which would test for an effect of sentence-level constraint (*correct* vs. *incorrect* sentences). Condition examples are shown in Table 7 below. All targets were repeated across all conditions, amounting to 400 experimental trials. We further retrieved stimuli for 12 practice trials, which were not included in the final data analyses. All trials were presented in randomized order.

**Table 7:** Experiment 2 condition examples.

	*Noun target*	*Verb target*
*Congruent*	cops rack cops	hear went hear
*Incongruent*	been rack been	cops went cops
*Correct*	this rack fell	they went here
*Incorrect*	fell rack this	here went they

#### Apparatus and software

All apparatus and software was similar to those used in Experiment 1, albeit without the use of an eye-tracker.

#### Procedure

Participants were seated in a comfortable office chair in a dimly lit testing room. The distance from the participants’ eyes to the computer screen was 90 cm, so that every character space subtended 0.35 degrees of visual angle. Centralized vertical fixation bars were presented throughout the experiment. Every trial, a target stimulus with flanking words (separated by one character space from the target) was presented for 170 ms at the center of the screen, in between the fixation bars, after which participants had a maximum of 2300 ms to respond with a left (‘z’) or right (‘/’) button response (qwerty keyboard layout) whether the target was a noun or verb. Responses for ‘noun’ were always matched to the right (‘/’) button. After correct responses, a green dot was briefly shown; for incorrect responses, this was a red dot. Participants were offered a break halfway through the experiment. The duration of the experiment was approximately 20 minutes.

### Results

Trials where the response time (RT) was beyond 2.5 standard deviations from the mean (3.14% of all trials) were discarded. Only correctly answered trials were included in the analysis of RTs, leading to the exclusion of another 7.59% of trials. To analyze RTs, we again employed LMM models with items and participants as crossed random effects (including random intercepts and the by-participant random slope). Generalized (logistic) LMM models with by-item and by-participant random intercepts were used to analyze the error rates.

#### Congruent vs. incongruent flankers

RTs as well as error rates (Table 8) were significantly lower in the congruent flanker condition as compared to the incongruent flanker condition (see Table 9), supporting the hypothesis that lexical information can be gathered and integrated across multiple words simultaneously.

**Table 8:** Experiment 2 mean RTs (ms) and error rates.

	*RT*	*Error*
*Congruent* (cops rack cops)	500.46 (150.26)	.062 (.003)
*Incongruent* (been rack been)	520.51 (152.54)	.094 (.004)
*Correct* (this rack fell)	504.85 (147.10)	.075 (.003)
*Incorrect* (fell rack this)	505.83 (150.34)	.077 (.004)

Note: values in between parentheses indicate standard deviations.

**Table 9:** Analyses of RTs and error rates: congruent vs. incongruent flankers.

	*RT*			*Error*		
	*b*	*SE*	*t*	*b*	*SE*	*z*
*(Intercept)*	505.52	17.00	**29.73**	3.20	0.18	**17.96**
*Incongruent*	17.45	4.06	**4.30**	0.53	0.12	**4.32**

Note: ref.: congruent flankers. Significant values are shown in bold.

#### Correct vs. incorrect sentence flankers

Whereas we found a difference between the congruent vs. incongruent flanker conditions, there was no difference between the correct- and incorrect sentence conditions (Table 10).

**Table 10:** Analyses of RTs and error rates: correct vs. incorrect sentence flankers.

	*RT*			*Error*		
	*b*	*SE*	*t*	*b*	*SE*	*z*
*(Intercept)*	508.93	17.00	**29.93**	2.96	0.17	**17.09**
*Incorrect*	-0.27	4.04	-0.07	-0.02	0.12	-0.19

Note: ref.: correct sentence flankers. Significant values are shown in bold.

### Discussion

In Experiment 2 we set out to investigate whether syntactic processing may occur across multiple words in parallel. We assessed two scenarios, one of which assumes that information from multiple words would culminate into one syntactic signal (e.g. “this is a noun”, “this is a verb”). The other scenario assumes that the syntactic categorization of words is dependent on constraints at the sentence level, meaning that readers would be more likely to expect (and thus faster to recognize) a noun at position *n* when it is flanked by syntactically compatible words, such as an adjective at position *n-1* and a verb at position *n+1* (in English), as compared to incompatible words. The results of Experiment 2 support the first scenario, as we found significant differences in RTs and errors between the syntactically congruent and incongruent conditions, while no differences were found between the correct- and incorrect sentence conditions.

The current results support the idea that higher-order processing can occur for multiple words in parallel. Indeed, considering that the time it takes to recognize a word is in the range of 150–250 ms (e.g. [37]), we reckon that there could not have been abundant time to process the target and two flankers serially in the 170 ms that they were presented. It should further be noted that flankers may interfere rather than facilitate—even when the flankers are congruent with the target with respect to the task at hand (e.g., [38]). In a recent study, we found that target processing was faster in a no-flanker condition than in the congruent flanker condition [39]. This suggests that parafoveal stimuli invariably demand attentional resources, in principle leading to slower target recognition. Crucially, this does not affect the implications of the current results: the fact that syntactically congruent flankers interfered less than syntactically incongruent flankers, provides evidence that the syntactic information of these flankers was available during target processing.

While we found syntactic parafoveal-on-foveal information integration in Experiment 2, we did not find such an effect in Experiment 1. If anything, in Experiment 1 the fixation duration on the pre-target (*n-*1) was more likely to be increased with a syntactically similar preview (*n*), as compared to a syntactically different preview. As argued in the Introduction, a potential explanation for this discrepancy is that different tasks might engage different cognitive processes. Specifically, it could be that sentence reading engages the maintenance of a sentence-level representation in working memory, from where top-down feedback would ensure that various syntactic categories are mapped onto the different word positions available in the visual field, to optimize higher-order sentence comprehension. It is conceivable that such a mechanism is not required, and thus not engaged, in a single-word reading paradigm such as the flanker task used in Experiment 2. This would also explain why there was no difference between the conditions where the flankers and target formed a correct vs. an incorrect sentence. Thus, while multiple words can be lexically processed in parallel, it is possible that higher-order mechanisms prohibit the parafoveal-on-foveal integration of higher-order information in sentence reading.

One important issue remains for future research investigating parafoveal processing of syntactic information. That is the potentially asymmetrical nature of such processing, being stronger in the direction of reading (i.e., in the right visual field for the current study). Indeed, there is abundant evidence for asymmetrical processing in the parafovea as concerns various types of information (e.g. [40]) in line with the fact that the span of effective vision in reading extends further in the direction of reading (e.g., [41, 42]). The present study was not designed to address this issue, but we suspect that evidence for such asymmetrical processing of syntactic information in the parafovea will be conditioned by the task used to investigate this (i.e., sentence reading vs. flanker paradigm), as should become obvious from the comparison of processing involved in Experiments 1 and 2 of the present study in the following discussion.

## General discussion

Multiple lines of research have alluded to the possibility that lexical access can occur for parafoveal (upcoming) words in sentence reading, although supporting evidence has been scarce [16–19]. While these studies have focused on semantic parafoveal preview effects, in the current research we focused on syntactic parafoveal effects as an alternative type of higher-order processing.

In Experiment 1 we found that the recognition of a target word was facilitated by a syntactically related preview at the target location during the fixation on the pre-target, suggesting that the reading system extracts higher-order information from parafoveal words. We then set out to investigate whether higher-order parafoveal word processing can take place *during* or rather *after* foveal word processing in Experiment 2, touching upon the highly debated question whether lexical processing occurs serially or rather across multiple words in parallel (e.g. [32, 33]). Multiple lines of research have shown that words can indeed be processed in parallel, by establishing *parafoveal-on-foveal* effects rather than parafoveal preview effects, but these effects were mainly of a sub-lexical orthographic nature (e.g. faster recognition of *n* due to orthographically related *n+1*; see [8–13]). Higher-order parallel processing has been more controversial; for instance, Angele et al. did not find parafoveal-on-foveal facilitation with semantically related stimuli ([8]; but see also [11]). As it turned out, in Experiment 2 we found evidence in support of the idea that multiple words can be syntactically processed in parallel, as the syntactic categorization of (foveal) target words was facilitated by syntactically congruent flanking words. Meanwhile, we did not find that words were categorized faster when they were syntactically compatible (i.e., formed a correct sentence) with flanking words.

There is an apparent contradiction between the results of Experiment 1 and Experiment 2 that needs addressing. While we found that word processing was facilitated by syntactically similar words in the parafovea in Experiment 2, this was not the case in Experiment 1. Specifically, we did not find that fixation durations on the pre-target were decreased with a syntactically similar preview, (i.e., the incongruent condition). On the contrary, fixation durations on the pre-target were numerically increased when it was followed by a word of the same syntactic type (see also [13]), alongside an increased fixation rate on the target, suggesting that the reading process was perturbed by the upcoming word. This is most likely due to the fact that word processing is influenced by sentence-level constraints in sentence reading, the underlying mechanisms of which are not engaged in a single-word reading task.

**Fig 2:**
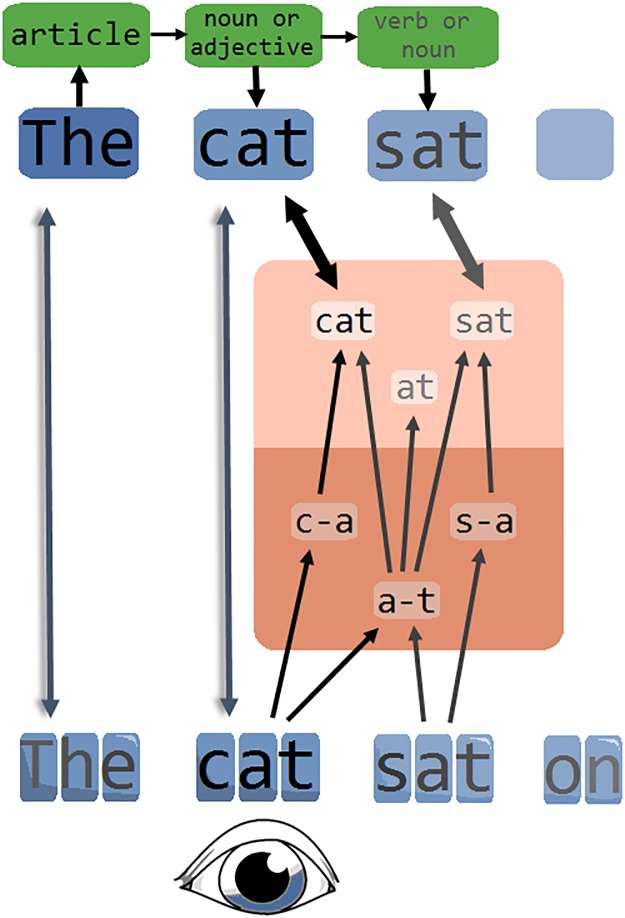
Our conceptualization of the reading system. Sub-lexical orthographic information is gathered across multiple words, with stronger activation of letters in the fovea (here *‘cat’*) than letters in the parafovea. Sub-lexical information activates word representations and, importantly, parafoveal information may help to activate the word representation belonging to the fovea if there is orthographic overlap, accounting for the orthographic parafoveal-on-foveal effects reported in the literature. Activated word representations are projected onto a plausible location in a spatiotopic representation, based on visual features such as word length and shape. From here, recognized words append to a sentence-level representation that follows syntactic rules: for instance, if word *n* is recognized as an article, word *n+1* is expected to be a noun or adjective (in English). Feedback from the syntactic level to the individual word positions constrains the recognition process while allowing for the simultaneous recognition of multiple words.

In this scenario, we argue that readers generally process multiple words simultaneously, with each activated word form also activating higher-order semantic and syntactic features (see Fig 2). In our flanker paradigm of Experiment 2, this resulted in the integration of higher-order information across foveal and parafoveal stimuli, such that parafoveal words influenced the decision about the foveally presented target word. During sentence reading, however, activated word forms would append to a sentence-level representation, from where feedback to individual word positions would constrain the recognition process for these words (e.g. through mapping various syntactic categories onto the multiple word positions available in the visual field).

Importantly, this scenario implies that readers are able to keep track of multiple words separately, explaining why previous research has not managed to establish higher-order parafoveal-on-foveal effects in sentence reading. If upcoming words produce a mismatch, for example because they are of an impossible grammatical category (as in the incongruent condition of Experiment 1), the recognition process would be slowed. Indeed, the results of Experiment 1 showed that target words were fixated longer and more often after an incongruent preview.

In sum, we propose that multiple words can be processed in parallel beyond the sub-lexical level, leading to higher-order parafoveal-foveal integration when readers are set out to recognize no more than one word (Experiment 2). During sentence reading, however, sentence-level feedback to individual word positions would constrain the recognition of these words, counteracting integrative effects (e.g. faster syntactic categorization due to syntactically congruent adjacent words) and thus explaining why higher-order parafoveal-on-foveal effects in sentence reading have been elusive.
